# Tissue inhibitor of metalloproteinase-1 stimulates proliferation of human cancer cells by inhibiting a metalloproteinase

**DOI:** 10.1038/sj.bjc.6601533

**Published:** 2004-01-20

**Authors:** J F Porter, S Shen, D T Denhardt

**Affiliations:** 1 The Graduate Program in Microbiology and Molecular Genetics, Department of Cell Biology and Neuroscience, Rutgers University, Nelson Laboratories, 604 Allison Road, Piscataway, NJ 08854, USA

**Keywords:** TIMP-1, metalloproteinases, proliferation, ERK, p38 kinase

## Abstract

TIMP-1, an ∼30 kDa glycosylated protein found predominantly in extracellular compartments, is involved in the regulation of a variety of developmental, remodelling, and pathological processes. One function of TIMP-1 is to inhibit certain members of a group of extracellular and cell surface enzymes known collectively as metalloproteinases (MP). These include the matrix metalloproteinases and the adamalysin-like disintegrin and metalloproteinases (ADAMs). Additional activities of TIMP-1 include potentiating the activity of erythroid precursors and stimulating proliferation of certain cancer cell lines. Published evidence suggests that the apparent proliferative action of TIMP-1 is independent of its MP-inhibitory activity; however, reports of a cell surface receptor for TIMP-1 have not been confirmed. We have utilised a baculovirus-based system to produce TIMP-1. Data presented here show that TIMP-1 and synthetic hydroxamate (GM6001) MP inhibitors stimulate proliferation and metabolic activity of MDA-MB-435 cancer cells with similar kinetics. An inactive hydroxamate derivative was ineffective. The TIMP-1-induced increase in proliferation and metabolic activity was not the consequence of the inhibition of apoptosis by TIMP-1 in the serum-free medium. These data taken together imply that the mechanism by which TIMP-1 enhances cell growth depends on its ability to inhibit a metalloproteinase, rather than to stimulate a cell surface receptor by a process independent of its MP-inhibitory activity. Inhibitors of extracellular regulated kinase (U0126) and p38 (SB203580), and to a lesser extent the phosphatidylinositol-3-kinase inhibitor LY294002, suppressed the action of TIMP-1. Assays for ERK1/2 and p38 showed that both were activated by TIMP-1 and GM6001. Mechanisms by which TIMP-1 might act to stimulate cell proliferation are described.

Tissue inhibitor of metalloproteinase-1 (TIMP-1) is an N-glycosylated, secreted protein that is found in plasma and other body fluids (Brew *et al*, 2000). Six disulphide bonds maintain the protein's structure and define two domains, an N-terminal inhibitory domain and a C-terminal regulatory domain. The disulphide bonds are a hindrance to the production of properly folded, active recombinant TIMP-1 in bacterial systems, although some successes have been reported (Cocuzzi *et al*, 1992; Kleine *et al*, 1993; Huang *et al*, 1996; Rajan *et al*, 1998; Davis *et al*, 2001). Here, we have utilised a baculovirus-based system to produce post-translationally modified and secreted TIMP-1 in insect cells.

The primary function of the TIMPs (four are known) is to inhibit various members of a group of ectoenzymes known as MPs, which include the adamalysin-like disintegrin and metalloproteinase (ADAM) and matrix metalloproteinase (MMP) families (Brew *et al*, 2000). MPs function in various physiological and pathophysiological processes, such as ovulation, embryogenesis, angiogenesis, wound healing and metastasis, that involve remodelling of the extracellular matrix (Chambers and Matrisian, 1997; Nagase and Woessner, 1999; Brew *et al*, 2000). TIMP-1 regulates these processes by virtue of its ability to inhibit MPs.

Paradoxically however, TIMP-1 expression has been found to be elevated in certain malignancies, and high levels of TIMP-1 expression in tumours, by either tumour or stromal cells, are predictive of poor patient prognosis with regard both to the length of the disease-free interval and to survival rate (Ree *et al*, 1997; McCarthy *et al*, 1999; Nakopoulou *et al*, 2002). Therefore, if TIMP-1 can inhibit MPs and tumorigenicity, as has been shown for example in studies with murine melanoma cells (Walther and Denhardt, 1996), then why is increased expression in patients with cancer linked to a poor prognosis (Denhardt, 2000)?

An answer to this question was suggested by the studies of Hayakawa *et al* (1992) showing that TIMP-1 could promote the growth of a variety of normal and transformed cells, a property reminiscent of its erythroid potentiating activity (Golde *et al*, 1980; Docherty *et al*, 1985; Avalos *et al*, 1988). Additionally, two breast cancer cell lines, MCF-7 and BC-61, have been shown to respond to TIMP-1 by an increase in proliferation. MCF-7 cells that were grown in medium containing foetal calf serum immunodepleted of TIMP-1 grew less well than cells in the same medium supplemented with TIMP-1 (Hayakawa *et al*, 1992).

Growth of BC-61 cells was stimulated by TIMP-1 in a dose-dependent fashion, and there was an increase in protein tyrosine phosphorylation; these cells expressed an 80-kDa transmembrane protein that could bind TIMP-1 both *in vivo* and *in vitro* (Luparello *et al*, 1999). In those cancers associated with high levels of TIMP-1 expression, it appears that TIMP-1 drives tumour progression as the result of its ability to stimulate proliferation. Interestingly, the MP-inhibitory activity of TIMP-1 and its growth-promoting function have been reported to be independent of one another (Hayakawa *et al*, 1994; Chesler *et al*, 1995), suggesting that TIMP-1 may have a second mode of action having nothing to do with its ability to inhibit MPs (Baker *et al*, 2002). That is the issue addressed in this paper. Evidence presented here strongly suggests that the proliferation caused by TIMP-1 treatment of MDA-MB-435 cells is the result of TIMP-1 inhibiting one or more MPs. Additionally, our results show that the growth stimulus results from the activation of MEK, p38, and to a lesser extent PI3K, in cells treated with either TIMP-1 or a synthetic MMP inhibitor.

## MATERIALS AND METHODS

### TIMP-1 production and purification

The entire cDNA, including the signal sequence, encoding human TIMP-1 (a generous gift of Dr Dylan Edwards, University of East Anglia, UK) was subcloned into the pIZ/V5-His plasmid in frame with the C-terminal histidine tag. BTI-TN-5B1-4 insect cells (*Trichoplusia ni*, the cabbage looper, grown in Express Five Serum-Free Medium) were then transfected with the pIZ/hTIMP-1V5-His plasmid using CellFECTIN (all products obtained from Invitrogen, Carlsbad, CA, USA) according to the manufacturer's instructions. Transfected cells were selected by addition of Zeocin (from Invitrogen, Carlsbad, CA, USA) to the medium at a concentration of 600 *μ*g ml^−1^. Pools of clones were isolated and conditioned medium was examined by Western blot analysis for the presence of human TIMP-1. The medium conditioned by these cells can be collected from cultures maintained for up to 2 months. The protein was purified from the conditioned medium with use of a nickel chelating resin according to the manufacturer's instructions (Novagen, Madison, WI, USA). The eluted fractions were analysed on Western blots for the presence of human TIMP-1. The fractions containing TIMP-1 were pooled and dialysed against 20 mM PIPES (Piperazine-*N*,*N*′-bis-[2-ethanesulphonic acid]) (pH 6.0) and 50 mM NaCl. The dialysed sample was applied to a sulphonylpropyl cation exchange resin and eluted using 1.0 M NaCl buffer. The eluted fractions were tested by OD_280_ for the presence of protein and the fractions at the peak were pooled and concentrated using a Centriprep concentrator from Amicon (Beverly, MA, USA). The concentrated sample was then electrophoresed on a 15% SDS–PAGE gel using a nonreducing loading buffer (Leber and Balkwill, 1997). A small portion of the gel was stained with Coomassie Brilliant Blue R-250 (Sigma, St Louis, MO, USA). The band corresponding to the molecular weight of human TIMP-1 was excised and the gel slice was cut into cubes of about 1 mm^3^ and electroeluted in a 10 000 MWCO dialysis bag and 10 ml of TAE buffer (40 mM Tris-acetate and 2 mM EDTA, pH=8.5) overnight at 45 V and 4°C. After a brief centrifugation to remove gel fragments, the supernatant was lyophilised and resuspended in 1 ml of 250 mM NaCl and 20% glycerol. The protein suspension was then filtered using a 0.22-*μ*m filter and the protein was quantified using OD_280_ and an extinction coefficient of 1 mg ml^−1^ (Gill and Hippel, 1989).

The results of a typical purification procedure starting with 400 ml of medium conditioned by insect cells permanently transfected with a TIMP-1-expressing vector are shown in Table 1

**Table 1:** Protein purification table for recombinant human TIMP-1 from a baculoviral-based system

**Step**	**Total protein (mg)**	**Percent recovered protein**	**Total activity (U)**	**Percent recovered activity**	**Specific activity (U mg^−1^)**
Medium	355.6	100	4597	100	12.93
Nickel column	8.4	2.4	1822	39.6	216.9
Sulphopropyl column	2.9	0.82	1007	21.9	347.2
Electroeluted	0.424	0.12	712.7	15.5	5525

Data are from a typical recombinant human TIMP-1 protein purification starting with 400 ml of medium conditioned by permanently transfected insect cells. Protein and activity levels were determined as described in Materials and Methods.

**Figure 1:**
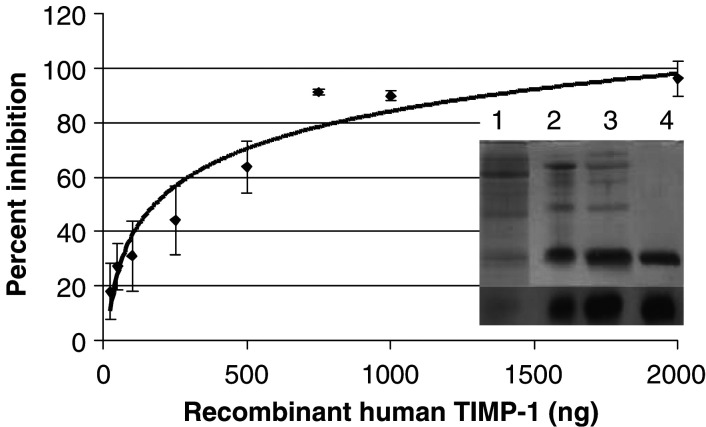
Inhibition of MMP-1 by purified TIMP-1 with inset figure of silver-stained SDS–PAGE and Western blot of purification steps for TIMP-1. Concentration-dependent inhibition of human fibroblast collagenase activity by purified human TIMP-1. The TIMP-1 concentration of 750 ng corresponds to a 1 : 1 molar ratio of enzyme to inhibitor. The inset consists of two parts; the top part is a silver stain of the fractions from each step in the purification of hTIMP-1. Lane 1 contains medium conditioned by transfected insect cells; lane 2 contains pooled fractions from a nickel column; lane 3 contains pooled fractions from an SP-cation exchange column; and lane 4 shows the electroeluted human TIMP-1. The bottom part is a Western blot of the same fractions showing a single band corresponding to hTIMP-1.

. The three-step purification procedure yielded approximately 1 mg of pure TIMP-1 (MW ∼30 kDa) per litre of conditioned medium. The purity (>95%) of the TIMP-1 preparation was assessed by Western blotting and silver staining of an SDS–PAGE gel (inset in Figure 1

). A collagenase inhibition assay, detailed in the next paragraph, was used to confirm the MMP-inhibiting activity of the purified TIMP-1. Figure 1 shows the result of a typical inhibition assay. Maximum inhibition occurred at 750 ng of TIMP-1, which corresponds to an approximate 1 : 1 molar ratio of TIMP-1 to active MMP-1 (MW ∼43 kDa), suggesting that the majority of both proteins in the preparations were functional.

### TIMP-1 activity assay

The MP inhibitory activity of the purified recombinant TIMP-1 was measured using human fibroblast collagenase (MMP-1, a generous gift of Dr Howard Welgus, Washington University School of Medicine, St Louis, MO, USA) and fluorescein-labelled collagen type I (Molecular Bioprobes, Eugene, OR, USA). An optimisation was done to determine the optimal amount of MMP-1 and fluorescein-labelled collagen for the activity assay. (Data not shown.) Briefly, differing amounts of purified TIMP-1 were mixed with 1 *μ*g MMP-1 in collagenase buffer (50 mM Tris-Cl pH 7.4, 0.2 M NaCl, 5 mM CaCl_2_ and 0.05% Brij-35) in a total volume of 95 *μ*l. The mixture was allowed to incubate for 1 h at room temperature to allow TIMP-1 and MMP-1 to associate. Following this incubation, 2.5 *μ*g of fluorescein-labelled collagen type I (Molecular Bioprobes), in 5 *μ*l, was added to each reaction. Fluorescence was measured using a Packard FluoroCount immediately after the addition of the collagen. The reactions were incubated in the dark at room temperature overnight. The next day a second fluorescent reading was taken and the first reading was subtracted from the second reading. The result was compared to a positive and negative control for collagen digestion to determine the amount of inhibition of MMP-1 by TIMP-1.

### Cell growth assays

Alamar Blue dye was purchased from Biosource International Inc. (Camarillo, CA, USA) and the manufacturer's instructions were followed to complete the assay (Ahmed *et al*, 1994; Nakayama *et al*, 1997). Briefly, MDA-MB-435 cells were plated in a 24-well dish in *α*MEM supplemented with 10% foetal bovine serum, 2 mM glutamine, 50 U ml^−1^ penicillin, and 50 *μ*g ml^−1^ streptomycin at a cell concentration of 15 000 cells well^−1^. (All cell culture media were obtained from Invitrogen.) Cells were allowed to attach overnight and the medium was replaced with *α*MEM supplemented with 1% bovine serum albumin (Sigma cell culture grade BSA, Catalog # A1933), 2 mM glutamine, 50 U ml^−1^ penicillin, 50 *μ*g ml^−1^ streptomycin and 10% Alamar Blue dye; the desired amount of TIMP-1 or synthetic inhibitor was also added. In experiments using a signal transduction inhibitor, the appropriate amount of signal transduction inhibitor was added and an initial incubation at 37°C and 5% CO_2_ for 1 h was done before the addition of TIMP-1. A Packard FluoroCount instrument was used to determine the amount of Alamar Blue reduced by measuring the fluorescence of the reaction mixture (excitation 530 nm, emission 590 nm).

Tritiated thymidine incorporation was quantified as described (Rittling *et al*, 2002). Briefly, cells were plated in complete medium in a 24-well dish at a concentration of 15 000 cells well^−1^ and allowed to attach overnight. The next day the wells were rinsed 3 × with phosphate-buffered saline (PBS); serum-free medium with 1% BSA was then added along with the appropriate amounts of TIMP-1 or synthetic inhibitor. The cells were then labelled, after incubation for differing time periods, with [^3^H]thymidine (1 *μ*Ci ml^−1^ of medium) for 5 h. The medium was then removed, the cells were rinsed once in ice-cold PBS and the cells in each well lysed with 500 *μ*l of 7% trichloroacetic acid on ice for 30 min. The plate was centrifuged at approximately 500 **g**, the supernatant removed, and the precipitate solubilised in 200 *μ*l of 0.5 M NaOH and 0.5% SDS. Radioactivity, typically ranging from 10^4^ to 10^5^ c.p.m. sample^−1^ in the solubilised precipitate, was determined with a Beckman scintillation spectrometer.

### Apoptosis assay

The apoptosis assay was performed using the Cell Death Detection ELISA Plus kit purchased from Roche-Applied Science (Penzberg, Germany, Catalog # 1 774 425). The manufacturer's protocol was followed to determine the level of apoptosis in MDA-MB-435 cells untreated or treated with TIMP-1 or synthetic inhibitor. Briefly, MDA-MB-435 cells were seeded into a 24-well dish at a concentration of 1.5 × 10^4^ cells well^−1^ and allowed to attach overnight. The next day the wells were rinsed 3 × with PBS and 1 ml of *α*MEM with 1%BSA was added to each well, followed immediately by the addition of TIMP-1, synthetic inhibitor, or the PBS vehicle. The cells were incubated for 24 h at 37°C with 5% CO_2_. Then the plate was centrifuged and the supernatant was carefully removed. The cell pellet was placed into 200 *μ*l of lysis buffer provided by the manufacturer for 30 min, after which time it was centrifuged. Aliquots of the supernatant (20 *μ*l) were used in an ELISA with anti-DNA and antihistone antibodies to detect the presence cytoplasmic nucleosomes. Advantages of this assay include the lack of subjectivity in interpreting the results, and further that it is sensitive enough to detect as few as 300 apoptotic cells.

### Synthetic MP inhibitors

GM6001 (Galardin, Ilomastat) and its inactive analog (*N*-*t*-butoxycarbonyl-L-leucyl-L-tryptophan methylamide) were obtained from Calbiochem (San Diego, CA, USA). BB94 (Batimastat) was a generous gift of Dr Dylan Edwards, University of East Anglia, UK.

### Signal transduction inhibitors

Signal transduction inhibitors (U0126, SB203580, LY294002, genistein, and H-9) were obtained from Tocris Cookson Inc. (Ellisville, MO, USA).

### ERK1/2 (p44/42) and p38 kinase assays

Kinase assay kits for both ERK1/2 (p44/42) and p38 were purchased from Cell Signaling Technologies, Beverly, MA, USA. The manufacturer's protocol for the assays was followed. Briefly, 4 × 10^5^ MDA-MB-435 cells were plated in 10-cm dishes and allowed to attach overnight. The next day the cells were rinsed 3 × with PBS and 5 ml of serum-free *α*MEM with 1% BSA was added and the cells were serum-starved overnight. The next day the cells were again rinsed 3 × with PBS and 5 ml of fresh *α*MEM+BSA medium with either TIMP-1, the synthetic hydroxamate inhibitor, or PBS vehicle. The cells were then incubated at 37°C in 5% CO_2_ for 20–30 min. The cells were then harvested in lysis buffer (provided by the manufacturer) with a cell scraper. Protein from the cell lysate amounting to 200 *μ*g for each p44/42 assay and 400 *μ*g for each p38 assay was added to an immunoprecipitation reaction overnight at 4°C to precipitate active p44/42 and p38, respectively. Kinase activity in the immunoprecipitates was assessed with either Elk-1 or ATF-2 as substrate for p44/42 and p38, respectively. The reaction mixtures were then electrophoresed on a 12% SDS–PAGE gel, blotted and probed for phosphorylated Elk-1 or ATF-2 using antibodies specific for the phosphorylated protein. The intensities of the bands generated on films of the gels were then quantified (in the linear range) using Kodak 1D Image Analysis Software (Eastman Kodak Company, Rochester, NY, USA).

### Statistical analysis

Statistical analysis was done using the Student's *t*-test.

## RESULTS

### TIMP-1 stimulates cell proliferation

**Figure 2:**
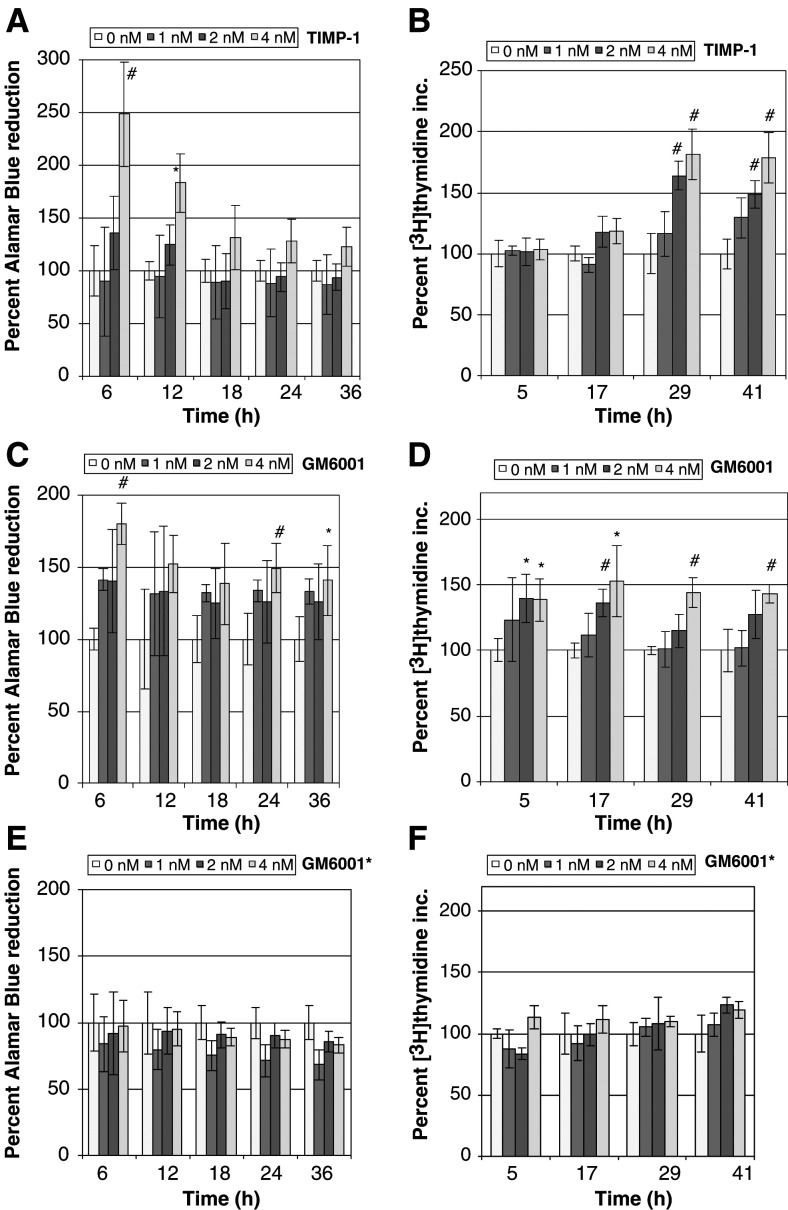
TIMP-1 and GM6001 stimulate anabolic activities in MB-MDA-435 cells in a time- and concentration-dependent manner. Panels (**A**) and (**B**) show, respectively, the enhancement of reduction of Alamar Blue and the stimulation of incorporation of [^3^H]thymidine by the concentration of TIMP-1 indicated in the inset. Panels (**C**) and (**D**) show, respectively, the enhancement of reduction of Alamar Blue and the stimulation of incorporation of [^3^H]thymidine by the concentration of GM6001 indicated on the inset. Panels (**E**) and (**F**) show, respectively, the lack of enhancement of reduction of Alamar Blue and no stimulation of incorporation of [^3^H]thymidine by the concentration of inactive GM6001 derivative (GM6001^*^). In the panels representing the Alamar Blue assays, the amount of Alamar Blue reduced in each sample was normalised to the untreated control (100% represents 2 × 10^3^–2 × 10^4^ RFU). Each bar represents the average of four samples. In the panels representing the [^3^H]thymidine incorporation cells were treated with or without TIMP-1 or synthetic inhibitors and pulsed for 5 h with [^3^H]thymidine. (Time on the abscissa is the time the 5-h pulse with [^3^H]thymidine was terminated.) The amount of [^3^H]thymidine incorporated in each sample was normalised to the untreated control (100% represents 2 × 10^4^–4 × 10^4^ c.p.m.). Each bar represents the average of four samples. Statistical significance was calculated using the Student's *t*-test; ^*^represents a *P*-value of 0.05 or less and # represents a *P*-value <0.01; RFU: relative fluorescence unit.

As noted in the introduction, TIMP-1 stimulates the growth of several transformed cell lines. We have used two different strategies to extend these studies. In one study, we assessed metabolic activity while in the second, we measured the rate of DNA synthesis, in both cases as a function of TIMP-1 concentration and exposure time. Figure 2A and B

show that TIMP-1 stimulated MDA-MB-435 by both criteria. Although generally considered a breast carcinoma line, recent evidence based on gene expression profiling has suggested that MDA-MB-435 may have been derived from an occult melanoma metastatic to the breast (Ellison *et al*, 2002). In Figure 2A, the respiratory rate of the cells treated with TIMP-1 increased by approximately 2.5-fold within the first 6 h of incubation in comparison with the untreated cells. Alamar Blue is reduced in proportion to mitochondrial respiration, and is thus an indicator of the overall rate of anabolic activity in the cell culture. In Figure 2B, the amount of DNA synthesis as measured by [^3^H]thymidine incorporation increased by almost two-fold 24 h after incubation with 4 nM TIMP-1 in comparison with the untreated cells, suggestive of a substantial increase in the rate of DNA replication. The concentration at which TIMP-1 caused maximal stimulation was 4 nM, in agreement with previous studies (Hayakawa *et al*, 1992, 1994; Yamashita *et al*, 1996). (Above 4 nM there was no further stimulation of proliferation or metabolic activity, data not shown.) These actions of TIMP-1 are unlikely to be the consequences of endotoxin contamination both because of the nonbacterial source of the protein and because an endotoxin assay (Sigma E-Toxate kit) revealed no evidence of endotoxin.

Previous literature reports suggest that the proliferation caused by TIMP-1 occurs through its interaction with a putative cell surface receptor and not through its interaction with an MP (Hayakawa *et al*, 1994; Chesler *et al*, 1995). In order to better address this question, we asked whether the synthetic MP inhibitor GM6001 (also known as Ilomastat or Galardin) and also an inactive derivative of GM6001 as a control, could similarly stimulate metabolic activity and DNA synthesis. GM6001 has been reported to inhibit MMP-1, -2, -3, -8, and -9 (Galardy *et al*, 1994a,1994b). As can be seen in Figure 2C, 4 nM GM6001 caused an 80% increase in the respiratory rate of MDA-MB-435 cells when treated for 6 h. Figure 2D shows a 50% increase in [^3^H]thymidine incorporation in cells incubated with GM6001 for 12 h. Very similar results (not shown) were obtained with BB94, which can inhibit MMP-1, -2, -3, -7, -9, and -13 (Brown, 1994). At concentrations above 4 nM, both of these inhibitors were unable to increase further either the respiratory rate or [^3^H]thymidine incorporation (data not shown). In comparison with TIMP-1, GM6001 was somewhat less effective at stimulating Alamar Blue reduction, but more effective at stimulating DNA synthesis, particularly in the first 12 h. Simultaneous exposure of the cells to TIMP-1 and GM6001 did not reveal evidence for an additive effect (data not shown).

**Figure 3:**
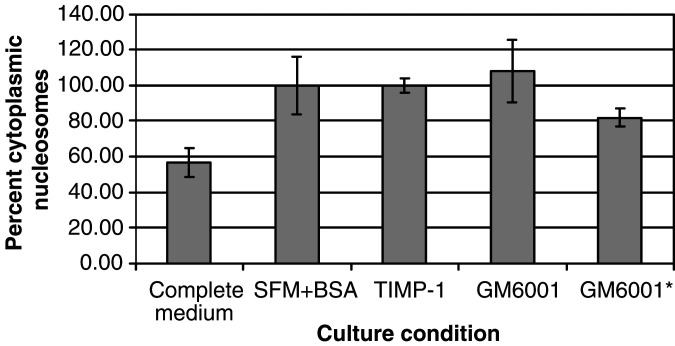
Apoptosis levels of differentially treated MDA-MB-435 cells as measured by cytoplasmic nucleosome detection. The histogram shows the percent of cytoplasmic nucleosomes detected in differentially treated MDA-MB-435 cells normalised to serum-free medium plus 1%BSA. The assay was done as described in the Materials and Methods section. Cells treated with TIMP-1, GM6001, or GM6001^*^ (all were added at a concentration of 4 nM) were all grown in serum-free medium with 1%BSA. There is no statistically significant difference in the level of apoptosis as measured by cytoplasmic nucleosome accumulation between cells grown in serum-free medium with 1%BSA and cells grown in the same medium with TIMP-1, GM6001, or GM6001^*^ added. (100% represents an OD_405_=0.45).

To determine if the stimulation seen by the synthetic MP inhibitors was dependent upon their ability to inhibit MPs, we tested an inactive derivative of GM6001 (GM6001^*^) (Jung *et al*, 2002). Figure 2E and F shows that the inactive derivative (GM6001^*^) had no effect either on the respiratory rate or [^3^H]thymidine incorporation, confirming that the growth-promoting activity of GM6001 was dependent upon its ability to inhibit MPs. A sensitive apoptosis assay, shown in Figure 3

, suggested that the increase in metabolic activity and DNA synthesis in the cultures exposed to TIMP-1 was not simply the result of a reduced amount of apoptosis.

### Stimulation of growth by TIMP-1 occurs through the MEK/ERK and p38 kinase pathways

In BC-61 cells exposed to TIMP-1 tyrosine-phosphorylated proteins were shown to increase in abundance (Luparello *et al*, 1999). Additionally, Wang *et al* (2002) have reported an increase in Ras-GTP complex formation in MG63 human osteosarcoma cells treated with TIMP-1. To determine in our studies what signal transduction pathway(s) is/are critical to TIMP-1-induced signalling, we employed several specific signal transduction inhibitors. U0126 (a MEK inhibitor), SB203580 (a p38 kinase inhibitor) and LY294002 (a PI3-kinase inhibitor) were the specific signal transduction inhibitors used to shed light on the cell signalling pathways involved (Vlahos *et al*, 1994; Cuenda *et al*, 1995; Favata *et al*, 1998; Davies *et al*, 2000). Both MEK and p38 kinase have been shown to increase cellular proliferation upon activation (Neve *et al*, 2002; Royuela *et al*, 2002). PI3-kinase has been shown to inhibit apoptosis and also cause proliferation upon activation (Neve *et al*, 2002).

**Figure 4:**
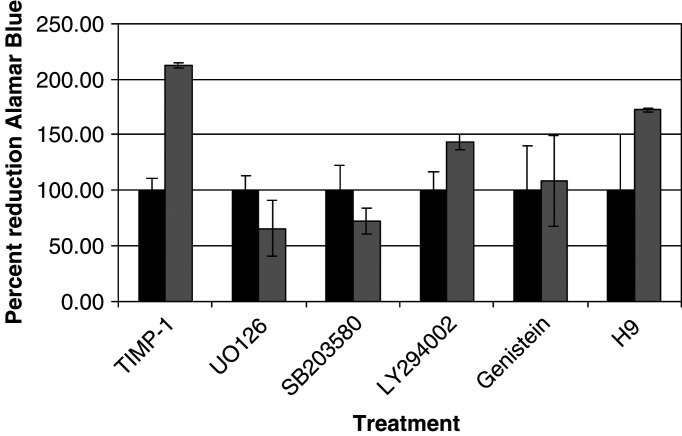
Action of signal transduction inhibitors on the ability of TIMP-1 to stimulate mitochondrial respiration. Signal transduction inhibitors were added (LY294002, 50 *μ*M; H9, 100 *μ*M, genestein, 10 *μ*M; SB203580, 10 *μ*M and U0126, 50 *μ*M) and incubated with the cells for 1 h prior to TIMP-1 addition. The Alamar Blue assay was done as described in the Materials and Methods section. The black bars show the metabolic activity of cells in the presence of the indicated signal transduction inhibitor alone; the grey bars show the activity when TIMP-1 is present. All cultures were incubated for 6 h from the time of TIMP-1 addition. The leftmost pair of bars in the graph show the activity in the absence of any signal transduction inhibitor. The extent of Alamar Blue reduction in the presence of the different signal transduction inhibitors did not differ by more than 10% from the control lacking any inhibition.

Additionally, two general signal transduction inhibitors were used: Genistein (an inhibitor of protein tyrosine kinases) and H-9 (an inhibitor of protein kinases including protein kinases A, C and G, calmodulin kinase II, and casein kinases I and II) (Akiyama *et al*, 1987; Flickinger, 1988). Activation of PKC has been shown to contribute to cancer cell proliferation (Jarzabek *et al*, 2002). Data generated from the signal transduction inhibitor experiments are summarised in Figure 4

. The two bars at the left show that the addition of TIMP-1 doubled the amount of Alarmar Blue reduction in the first 6 h under these conditions. The rightmost six bars represent cells treated with the indicated signal transduction inhibitor and 4 nM TIMP-1. U0126, SB203580 and Genistein all completely inhibited the increase in metabolic rate seen at 6 h when TIMP-1 alone was added to the cells. LY294002 and H9 appeared to inhibit partially the metabolic rate increase seen when the cells were treated with TIMP-1 alone.

**Figure 5:**
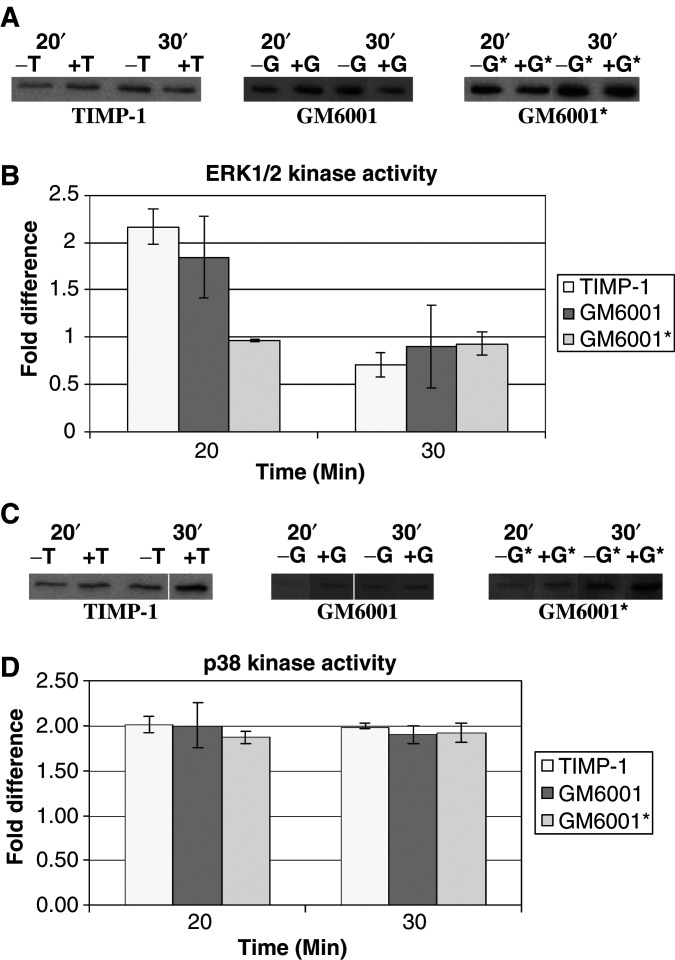
ERK activity is transiently increased at 20 min and p38 kinase activity is increased at 20 and 30 min post-treatment with TIMP-1 or GM6001. Kinase activity assays were performed as described in Materials and Methods. The Western blots shown in panel (**A**) indicate the level of ERK activity associated with MDA-MB-435 cells. The bands are phosphorylated recombinant Elk-1, which was phosphorylated by ERK immunoprecipitated from MDA-MB-435 cells. The first two lanes of each treatment set represent samples taken at 20 min post-treatment and the second two lanes of each treatment set represent samples taken at 30 min. Control and treated samples alternate as indicated in the figure. Densitometry data for the ERK activity assay are shown in panel (**B**). They indicate a two-fold increase in ERK activity for both TIMP-1 and GM6001 at 20 min relative to an untreated control. Panel (**C**) shows the level of p38 kinase activity as measured by recombinant ATF-2 phosphorylated by immunoprecipitated p38 kinase from MDA-MB-435 cells. Panel (**D**) shows densitometry data for p38 kinase assays. There is a clear two-fold increase in p38 kinase activity for all three treatments relative to an untreated control.

To confirm that the MEK/ERK and p38 kinase pathways are involved in TIMP-1 cell proliferation, ERK and p38 kinase activity assays were done on treated and untreated cells. The ERK assays are pictured in Figure 5A

; the band shown represents phosphorylated recombinant Elk-1, phosphorylated by immunoprecipitated ERK from MDA-MB-435 cancer cells. TIMP-1-treated cells exhibited a transient two-fold increase in ERK activity at 20 min as measured by the intensity of the phosphorylated Elk-1 (Figure 5B). By 30 min the ERK activity was back to baseline. Similarly, the cells treated with the synthetic MMP inhibitor GM6001, but not its inactive derivative, showed the same transient increase in ERK activity. The p38 kinase assays are shown in Figure 5C; the band shown represents phosphorylated recombinant ATF-2, phosphorylated by immunoprecipitated p38 kinase from MDA-MB-435 cells. TIMP-1, GM6001, and the inactive derivative of GM6001 all show the same effect on p38 kinase activity in the MDA-MB-435 cells. Activity of p38 kinase is ultimately increased in the treated cells by a factor of 2 by 30 min post-treatment as indicated by the densitometry data shown in Figure 5D. Similar results were obtained in a repeat experiment for both kinases.

## DISCUSSION

### TIMP-1 stimulates cell growth by inhibiting an MP

TIMP-1 has been shown to enhance proliferation of erythroid progenitors as well as certain transformed mammalian cell lines (Golde *et al*, 1980; Docherty *et al*, 1985; Hayakawa *et al*, 1992). Since TIMP-1 was first described as erythroid potentiating activity [EPA – (Golde *et al*, 1980)], several studies have reported the existence of a putative EPA/TIMP-1 receptor (Avalos *et al*, 1988; Chesler *et al*, 1995; Luparello *et al*, 1999). However, none of these reports have been independently verified, and no receptor has been cloned. Some experiments have been interpreted as evidence that the inhibitory activity and the growth-promoting activity were independent functions of TIMP-1 (Hayakawa *et al*, 1994; Chesler *et al*, 1995). The publication by Chesler *et al* (1995) dealt with the erythroid potentiation activity measured in nucleated cells isolated from human peripheral blood. The critical observation was that TIMP-1 causes an increased level of differentiation in these cells, increasing the number of erythroid precursors, independent of its MMP inhibitory activity; an increase in the rate of cell proliferation was not documented. In the Hayakawa *et al* (1994) paper, TIMP-1 was denatured and the sulphydral groups were alkylated. This alkylated form lacked the ability to inhibit MMPs and yet could could stimulate DNA synthesis in Raji cells, albeit much less effectively than the unalkylated form. Neither of these reports is contradicted by the results reported here.

The data in Figure 2A and B suggests that TIMP-1 stimulates the metabolism and proliferation of the human breast cancer cells studied here through its ability to inhibit an MP. Two synthetic broad-spectrum MP inhibitors, GM6001 and Batimastat (data not shown for Batimastat) were found to augment cellular proliferation at about the same molar concentration as TIMP-1, albeit to a lesser extent. This growth stimulation was demonstrated using two separate anabolic assays based on two different principles, mitochondrial activity and DNA synthesis. Stimulation of metabolic activity preceded the increase in DNA synthesis. These data suggest that both TIMP-1 and the synthetic MP inhibitors act on the same target or group of targets.

To confirm that the action of the synthetic MP inhibitors depended on their ability to inhibit MPs, an inactive derivative was also studied (Figure 2E and F). This inactive derivative, although similar in structure to GM6001, has been shown to be inactive against the same MMPs that GM6001 inhibits (Jung *et al*, 2002). It was unable to stimulate Alamar Blue reduction or [^3^H]thymidine incorporation. Decreased apoptosis did not appear to be responsible for the increase in metabolic activity and DNA synthesis induced by TIMP-1 (Figure 3).

## Anabolic response to TIMP-1 depends on MEK/ERK and p38 kinase activity

In order to determine which signal transduction pathways were required for the response to TIMP-1, we employed both specific and general signal transduction inhibitors. Although several of them partially inhibited Alamar Blue reduction (noted in the legend to Figure 4), the effect on TIMP-1 signalling was quite unambiguous. The specific signal transduction inhibitors for MEK and p38 kinase both completely inhibited the TIMP-1 signal. Additionally, the general tyrosine phosphorylation inhibitor, Genistein, also abolished the TIMP-1 response. These data together strongly implicate MEK and p38 kinase signal transduction pathways in mediating the growth stimulus delivered by TIMP-1. Other signal transduction inhibitors were also tested. These included LY294002, a specific inhibitor of PI3-kinase and H-9, which targets PKC and several other protein kinases. Pathways utilising PI3-K and PKC did not contribute substantially to the metabolic response elicited by TIMP-1.

Activity assays for the ERK1/2 and p38 kinases showed that TIMP-1 and GM6001 activated ERK1/2 and p38 kinases with different kinetics over the 30 min time frame (Figure 5). Also, interestingly, while the inactive derivative of GM6001 lacked the ability to activate ERK1/2 and to elicit a metabolic response, it nevertheless appeared able to activate p38. These differences reveal surprising complexity in the response of the cell to these MP inhibitors. Nevertheless, these data together with the signal transduction data indicate that activation of both ERK1/2 and p38 kinases is necessary for TIMP-1 stimulated proliferation to occur in treated cells, and they are consistent with the hypothesis that the synthetic inhibitors are acting through the same pathways as TIMP-1.

### Mechanisms of TIMP-1 action

How can inhibition of an MP result in increased growth of a cell? Fowlkes and Winkler (2002) have recently reviewed how members of the metzincin family (MMPs, adamalysin-related proteinases) affect the availability of growth factors and cytokines. One possibility is that TIMP-1 prevents the degradation of a newly synthesised growth factor by a constitutively active MP, an ADAM for example. There are several examples of growth factors that MPs are capable of modifying, for example, FGF-R1, Pro-TGF-*β*2, and IGF/IGFBPs (McCawley and Matrisian, 2001). Another potential mechanism could be that TIMP-1 prevents the cleavage of a cell surface receptor that when activated by, for example, a ligand on another cell, stimulates proliferation. This mechanism relates to receptor shedding, a process used by some cells to regulate signal transduction pathways (Tokumaru *et al*, 2000; Chen *et al*, 2001). Some MPs are capable of cleaving some cell surface receptors (Smith *et al*, 1997; Lombard *et al*, 1998).

A final more speculative mechanism would be that TIMP-1 acts by binding to a membrane-type MMP and causing the activation of a signal transduction pathway through the cytoplasmic domain of the membrane-type MMP. This is more speculative in that the two MT-MMPs known to interact with TIMP-1 (MT4-MMP and MT6-MMP) are not known to be capable of stimulating an intracellular signalling pathway (Baker *et al*, 2002). In a real-time screen of MMP mRNA levels in these cells, mRNAs encoding MMP13 (collagenase 3) and MMP17 (MT4-MMP) were the most abundant (D Edwards, University of East Anglia, personal communication). This proposed pathway could, for example, lead to the upregulation of growth factor genes or the downregulation of growth inhibitor genes. The cytoplasmic domains of membrane-type MMPs appear to have some function in the regulation of the membrane-type MMPs themselves (Lehti *et al*, 2000, 2002). Further research is needed to elucidate the mechanism by which TIMP-1 ‘signals’ by inhibiting an MP. This information may be important for understanding why increased expression of TIMP-1 in some cancer patients is linked to a poor prognosis (Ree *et al*, 1997; McCarthy *et al*, 1999; Denhardt, 2000; Nakopoulou *et al*, 2002).
